# Geranylgeranoic acid, toll-like receptor 4, and pyroptosis-related signaling in hepatocellular carcinoma: a hypothesis-generating perspective

**DOI:** 10.3389/or.2026.1882953

**Published:** 2026-08-18

**Authors:** Yuki Tabata

**Affiliations:** Department of Nursing, Faculty of Nursing, Miyazaki Prefectural Nursing University, Miyazaki, Japan

**Keywords:** geranylgeranoic acid, hepatocellular carcinoma, inflammasome, inflammatory cell death, pyroptosis, Toll-like receptor 4

## Abstract

Hepatocellular carcinoma (HCC) develops in a chronically inflamed hepatic environment shaped by innate immune signaling, tissue injury, and microenvironmental remodeling. Toll-like receptor 4 (TLR4) signaling and pyroptosis are frequently discussed together in this context because both connect danger sensing to inflammatory output. However, marker-associated changes in TLR4 expression, inflammasome-related proteins, cytokine release, or lytic cell death do not necessarily establish a causally defined TLR4-to-pyroptosis pathway. This Perspective examines the unresolved interface between TLR4-associated inflammatory priming and pyroptosis-related execution in hepatoma-cell systems, with particular attention to the distinction between conceptual overlap and mechanistic continuity. I further discuss geranylgeranoic acid (GGA), an endogenous diterpenoid acid with reported anti-hepatoma activity, for which a functional contribution of TLR4-associated signaling has been reported in HuH-7 cells, although direct receptor engagement and generalizability across hepatoma models remain unestablished. Accordingly, GGA is considered a hypothesis-generating experimental probe to refine questions concerning receptor-complex modulation, membrane microenvironment remodeling, stress- or danger-signal-mediated amplification, and inflammatory cell-death susceptibility. Overall, the available evidence supports a cautious but experimentally tractable framework in which TLR4-associated signaling may contribute to inflammatory priming and, under permissive conditions, intersect with pyroptosis-related outcomes. Defining this interface will require direct tests of pathway dependence, temporal ordering, and execution-level validation.

## 1 Introduction

Hepatocellular carcinoma (HCC) develops within a chronically injured and inflamed hepatic environment shaped by viral hepatitis, metabolic dysfunction-associated steatotic liver disease (MASLD), fibrosis, cirrhosis, and gut-derived inflammatory signals (1–3). Accordingly, hepatocarcinogenesis should be interpreted not only as a tumor-cell-autonomous process, but also as a consequence of inflammatory signaling, stromal reactivity, and tissue remodeling in the diseased liver (2, 4, 5).

Among inflammation-related cell-death programs, pyroptosis has attracted attention because it links intracellular danger sensing to extracellular immune activation through membrane disruption and inflammatory mediator release (6). In HCC, however, pyroptosis is unlikely to be uniformly tumor-suppressive or tumor-promoting; depending on cellular context and disease stage, it may either eliminate damaged or transformed cells or amplify chronic inflammation, fibrogenesis, and tumor-supportive microenvironmental remodeling (7, 8).

**FIGURE 1:**
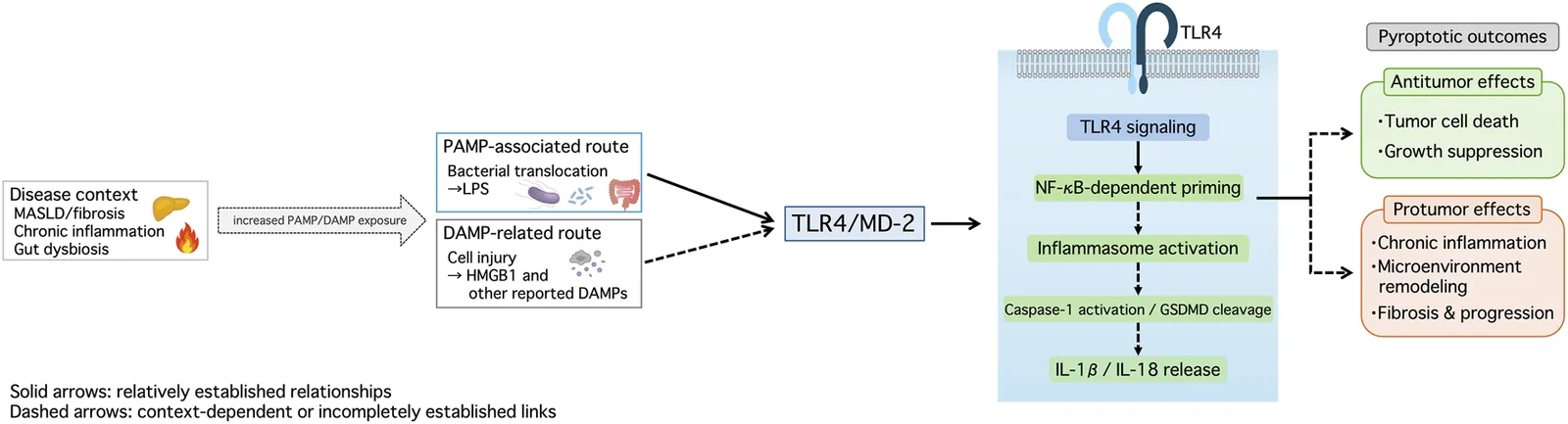
Context-dependent TLR4-associated inflammatory and pyroptosis-related signaling in hepatocellular carcinoma. Upstream inputs are separated into pathogen-associated molecular pattern (PAMP)- and damage-associated molecular pattern (DAMP)-related routes. Bacterial lipopolysaccharide (LPS), including LPS derived from gut microbial translocation, is shown as the prototypical PAMP recognized by the TLR4/MD-2 complex. Endogenous damage-related cues, including HMGB1 and other reported DAMPs, are shown as context-dependent inputs because their ability to engage TLR4 may depend on molecular state, extracellular cofactors, and biological context. MASLD/fibrosis, chronic inflammation, and gut dysbiosis are presented as disease contexts that may increase exposure to PAMPs or DAMPs rather than as direct TLR4 ligands. Cellular injury is shown as an upstream source of DAMP release. Downstream processes include NF-κB-associated priming, inflammasome activation, caspase-1/GSDMD involvement, and IL-1β/IL-18 release. Pyroptosis-related responses may exert antitumor or protumor effects depending on the biological context. Solid arrows indicate relatively established relationships, whereas dashed arrows indicate context-dependent, hypothetical, or incompletely established links.

Within this framework, Toll-like receptor 4 (TLR4) is of particular interest because it integrates microbial stimuli, endogenous danger signals, metabolic injury, and sterile inflammation in the context of the gut–liver axis (9–12). TLR4-associated signaling has been implicated in inflammatory priming, stellate-cell activation, macrophage modulation, and broader immune remodeling during hepatocarcinogenesis (13–15). However, its relationship to pyroptosis-related signaling in HCC remains incompletely defined. In particular, TLR4 activation, inflammasome-related marker changes, and pyroptosis-related phenotypes are often discussed within a common framework even when direct mechanistic continuity has not been established (7, 16). This distinction is important because inflammatory signaling, multiple cell-death programs, and microenvironmental remodeling frequently overlap in HCC. The conceptual relationship among upstream inflammatory cues, TLR4-associated signaling, and context-dependent pyroptosis-related outcomes is summarized in Figure 1.

This unresolved interface provides a rationale for reconsidering geranylgeranoic acid (GGA), an endogenous diterpenoid acid with reported anti-hepatoma activity (17–21). In HuH-7 cells, GGA-induced cell death and pyroptosis-related responses were attenuated by pharmacological TLR4 inhibition and siRNA-mediated TLR4 knockdown, supporting functional involvement of TLR4-associated signaling in this specific model (16). However, the proximal event connecting GGA exposure to TLR4-associated signaling remains unclear, and direct engagement of the TLR4/MD-2 complex has not been demonstrated (16, 22, 23). GGA should therefore not be positioned as a direct TLR4 ligand or as a universally established regulator of TLR4-dependent pyroptosis. Rather, this Perspective treats GGA as a hypothesis-generating experimental probe that may help clarify lipid-mediated, membrane-associated, or stress-linked upstream events converging on inflammatory cell-death susceptibility in HCC-related experimental systems. The aim is not to establish a closed TLR4–pyroptosis pathway, but to define an experimental framework for testing how GGA-related perturbations may intersect with TLR4-associated priming and pyroptosis-related outcomes.

**FIGURE 2:**
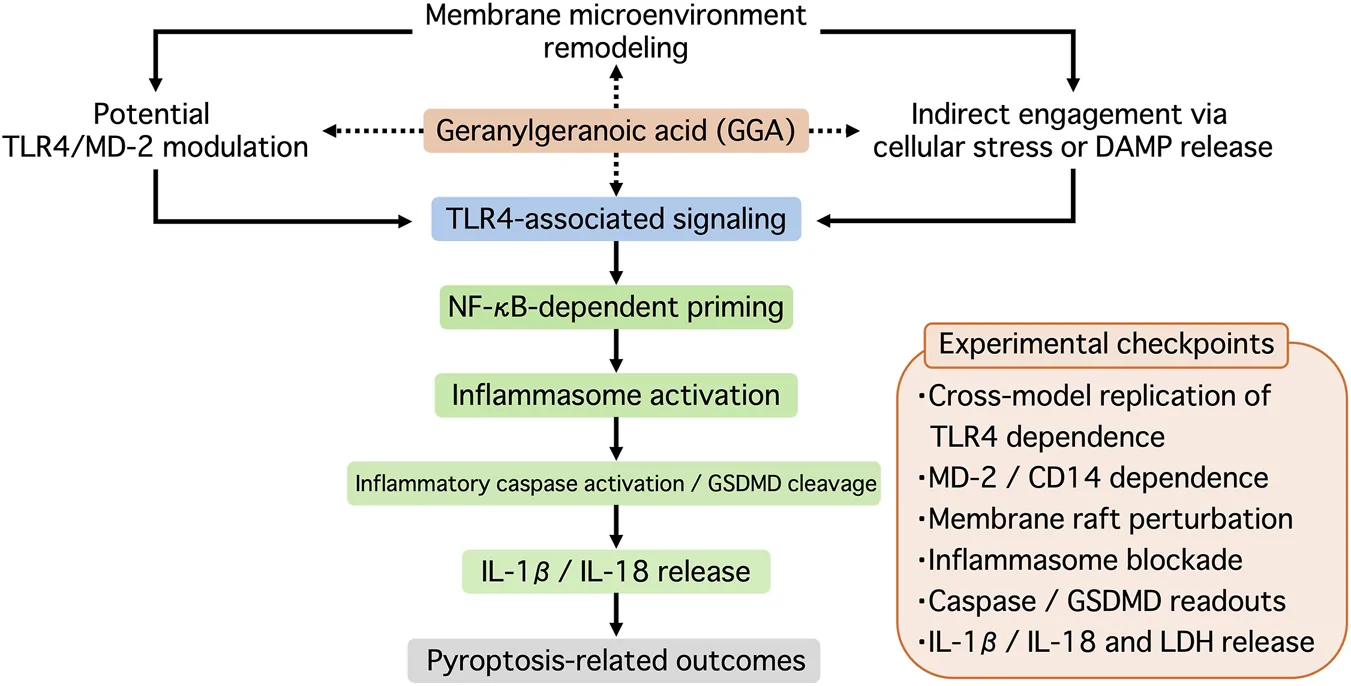
Geranylgeranoic acid as a hypothesis-generating probe for TLR4-associated inflammatory cell-death signaling in HCC. This schematic summarizes a working model in which geranylgeranoic acid (GGA) may serve as an experimental probe to examine unresolved upstream events linking lipid-associated perturbation to inflammatory cell-death susceptibility in hepatoma cells. GGA may influence this interface through potential modulation of the TLR4/MD-2 complex, membrane microenvironment remodeling, or indirect engagement via cellular stress and DAMP release. These upstream events may converge on TLR4-associated signaling, NF-κB-dependent priming, inflammasome activation, caspase-1/GSDMD involvement, IL-1β/IL-18 release, and pyroptosis-related outcomes under permissive conditions. Dashed arrows indicate hypothetical or incompletely established links that require experimental validation.

The manuscript is organized accordingly. Sections 2–4 develop the progression from upstream inflammatory inputs and TLR4-associated priming to context-dependent pyroptosis-related execution, as summarized in Figure 1. Sections 5, 6 then use Figure 2 to position GGA as a hypothesis-generating probe and to define the experimental steps required to resolve the proposed interface.

## 2 Upstream inputs to TLR4-associated signaling in HCC

TLR4 is a type I transmembrane pattern-recognition receptor (PRR) that functions together with MD-2 and accessory proteins to detect pathogen-associated molecular patterns (PAMPs) and selected damage-associated molecular patterns (DAMPs). Its canonical microbial agonist is lipopolysaccharide (LPS), particularly the lipid A moiety, from Gram-negative bacteria. LPS is captured by LPS-binding protein (LBP), transferred to CD14, and delivered to MD-2, thereby promoting TLR4/MD-2 dimerization and downstream signaling (24–28). In the liver, bacterial translocation across the gut–liver axis provides a relevant source of this PAMP. Sterile injury may additionally generate TLR4-active DAMPs, including the cytokine-active disulfide form of HMGB1 and reported extracellular-matrix-derived signals such as fibronectin-EDA (29–31). These endogenous inputs should not be treated as mechanistically equivalent to LPS, because their activity may depend on molecular state, co-receptors, or experimental preparation, and direct engagement of the TLR4/MD-2 complex is not equally established for all candidates (31, 32).

Representative experimental modulators help distinguish receptor activation from pathway inhibition. LPS and monophosphoryl lipid A (MPLA) are TLR4/MD-2 agonists; eritoran occupies MD-2 without inducing productive receptor activation, whereas TAK-242 (resatorvid) binds Cys747 in the cytoplasmic TLR4 domain and blocks adaptor-dependent signaling (10, 28, 32–34). TAK-242 is therefore more accurately termed a TLR4 signaling inhibitor than a ligand-binding antagonist. Cell-surface TLR4 primarily initiates TIRAP/MyD88-dependent NF-κB and MAPK priming, whereas CD14-dependent internalization enables TRAM/TRIF signaling (26, 27). In HCC, these pathways operate across hepatocytes, macrophage-lineage cells, hepatic stellate cells, and, in some settings, tumor cells (35–41). Their consequences remain dependent on ligand source, signal intensity, cell type, and disease model (12, 42–45). TLR4 is therefore an upstream coordinator of inflammatory priming and remodeling, rather than a direct executor of pyroptosis; its activation may establish a permissive state but does not alone demonstrate pyroptotic execution.

## 3 Pyroptosis pathways and cellular-context dependence in HCC

Pyroptosis is a lytic form of regulated cell death characterized by gasdermin-mediated membrane disruption, cell swelling, membrane rupture, and inflammatory mediator release (6, 46, 47). Because it links intracellular danger sensing to extracellular inflammatory signaling, pyroptosis has attracted attention in cancer biology at the interface of cell death, inflammation, and tissue-level immune responses. In the liver, its relevance is heightened by the chronically injured, inflamed, and regeneration-prone background in which HCC develops (7, 48). Under these conditions, inflammatory cell death may affect not only dying cells themselves, but also cytokine gradients, immune-cell recruitment, stromal activation, and fibrotic remodeling (49).

At the same time, the biological significance of pyroptosis in HCC remains context dependent (7, 50). Pyroptosis may limit tumor growth by eliminating damaged, transformed, or therapy-stressed cells, whereas repeated or dysregulated activation may sustain chronic inflammation, tissue injury, and tumor-supportive microenvironmental remodeling (7, 50, 51). Therefore, pyroptosis in HCC should not be inferred from marker expression alone. Increased inflammasome-related mediators, gasdermin-associated proteins, inflammatory cytokines, or lytic cell-death phenotypes may suggest pyroptosis-related signaling, but they do not necessarily demonstrate a complete and causally defined pyroptotic program (6, 52–54). This limitation is particularly relevant in tissue-level studies, where signals may reflect mixed contributions from tumor cells, hepatocytes, macrophages, and stromal compartments rather than a tumor-cell-autonomous process.

This heterogeneity creates an important interpretive problem. Findings from tumor tissues, macrophage-centered systems, fibrosis models, and hepatoma cell lines are all relevant, but they do not address the same biological question (7, 51, 55). Pyroptosis-related signaling may represent growth-limiting tumor-cell death in some settings, but in immune or stromal compartments it may also reinforce antitumor immunity or sustain chronic inflammatory support for tumor progression (51, 56). Moreover, hepatoma cells often display overlapping features of multiple stress and cell-death programs, making it difficult to identify pyroptosis as the exclusive or initiating mechanism on the basis of inflammatory markers alone (50, 57). Thus, the key issue is not simply whether pyroptosis-related machinery is present in HCC, but whether TLR4-associated priming can be causally linked to pyroptosis-related execution in defined hepatoma-cell systems.

## 4 Linking TLR4-associated priming to pyroptosis-related execution in hepatoma cells

A central question is whether TLR4-associated priming can be mechanistically linked to pyroptosis-related execution in hepatoma cells. Conceptually, such a connection is plausible because TLR4 senses microbial and endogenous danger signals, whereas pyroptosis requires a permissive state for inflammatory priming, inflammasome-related activation, and membrane-disruptive execution (6, 10). However, conceptual overlap should not be equated with direct mechanistic continuity. At present, the strongest support for a TLR4-to-pyroptosis relationship lies at the level of inflammatory priming. By promoting NF-κB-associated inflammatory programs, TLR4 may lower the threshold for downstream inflammasome engagement, inflammatory caspase activation, or gasdermin-dependent membrane disruption when secondary stress, membrane perturbation, or endogenous danger signaling is superimposed (58, 59). Thus, TLR4 need not function as a direct executor of pyroptosis to remain mechanistically relevant; its role may instead be to establish a permissive intracellular state for pyroptosis-related outcomes under defined conditions.

Noncanonical pyroptosis also merits separate consideration. Whereas activation of the canonical NLRP3–caspase-1 axis often requires a transcriptional priming step that can be provided by TLR4 signaling, cytosolic LPS is directly sensed by caspase-4 and caspase-5 in humans and by caspase-11 in mice; these inflammatory caspases cleave GSDMD and can drive lytic cell death (60). Accordingly, the noncanonical route should not be represented as a simple downstream continuation of cell-surface TLR4 signaling. In murine myeloid cells, however, TLR4–TRIF–type I interferon signaling can induce caspase-11 and thereby license responses to cytosolic LPS, providing a defined example of TLR4-dependent conditioning rather than direct pyroptotic execution (61). Whether an analogous relationship regulates caspase-4/5 in hepatoma cells, and whether LPS can access the cytosol in biologically relevant HCC settings, remain unresolved.

Other gasdermin-mediated routes further broaden the interpretation of lytic phenotypes in cancer cells. Caspase-3 can cleave GSDME, and caspase-8 can cleave GSDMD in specific stress or infection-related settings (62, 63). However, these pathways have not been functionally established as TLR4-dependent mechanisms in hepatoma cells. They should therefore be regarded as alternative mechanisms to be distinguished experimentally, rather than as evidence for a continuous TLR4-to-pyroptosis pathway.

TLR4 may also contribute through signal amplification rather than simple initiation. Cellular stress or damage may generate endogenous ligands or membrane alterations that reinforce TLR4-associated signaling, thereby promoting feed-forward inflammatory susceptibility and increasing the likelihood of pyroptosis-related outcomes (10, 29). This model is consistent with chronic liver injury, in which tissue damage, innate immune activation, and inflammatory amplification can become self-reinforcing (12, 64). In hepatoma cells, however, this remains more plausible than proven. Pyroptosis-related signaling is often inferred from increased TLR4 expression, NF-κB activation, inflammasome-related proteins, inflammatory cytokines, or lytic loss of viability (6, 65). These findings are suggestive, but they do not establish functional TLR4 dependence, gasdermin-mediated execution, or continuous causality from TLR4 activation to pyroptotic death. Thus, current evidence is relatively supportive for inflammatory priming, but more limited for downstream execution and temporal ordering.

At the same time, selected hepatoma studies suggest that this relationship can be experimentally tested in defined cellular contexts. For example, in HuH-7 cells, disruption of an HCC-associated regulatory piRNA increased TLR4 expression, activated the TLR4/NF-κB/NLRP3 pathway, and was accompanied by caspase-1 activation, cleaved GSDMD, LDH release, and pyroptosis-related membrane damage; importantly, downstream signaling was attenuated by TLR4 silencing (66). Although this represents a specific regulatory setting rather than a universal hepatoma mechanism, it provides a useful example in which receptor-associated signaling, inflammasome-related activation, and lytic execution can be connected more directly than in marker-based descriptive studies. However, hepatoma cell lines are biologically heterogeneous, differing in differentiation state, metabolic organization, basal inflammatory tone, and responsiveness to innate immune stimuli (67, 68). This variability cautions against defining a universal hepatoma-cell pyroptosis program from partial or model-specific findings.

These considerations influence whether TLR4-associated signaling is interpreted as a protumor inflammatory state, a latent lytic vulnerability, or a context-dependent transition between the two. If TLR4-associated priming does not progress to lytic execution, its significance may lie mainly in microenvironmental conditioning and chronic inflammatory support for tumor progression. If, however, it proceeds to caspase- and gasdermin-dependent membrane damage in selected hepatoma backgrounds, it may represent a more direct growth-limiting mechanism (6–8, 69). The most defensible interpretation is therefore that the contribution of TLR4-associated priming to pyroptosis-related execution in hepatoma cells remains plausible but incompletely resolved. Future studies should directly test whether TLR4 inhibition attenuates downstream inflammasome-related signaling; determine whether lytic membrane damage involves caspase-1 activation with GSDMD cleavage or caspase-4/5-dependent GSDMD cleavage (6, 16, 60); and establish whether the relevant events can be temporally ordered rather than occurring as parallel stress responses. In addition, pyroptosis-related injury may release DAMPs that secondarily engage TLR4-associated pathways in macrophages, Kupffer cells, or hepatic stellate cells, amplifying paracrine inflammatory crosstalk (70, 71). This unresolved upstream logic provides a rationale for considering GGA as an experimental probe to test whether receptor-level modulation, membrane reorganization, stress-mediated danger signaling, or their combination precedes pyroptosis-related outcomes in hepatoma cells.

## 5 GGA as a hypothesis-generating probe of the unresolved interface

Building on the unresolved interface defined above, geranylgeranoic acid (GGA) is an endogenous diterpenoid acid that is biosynthesized in mammalian cells and has reported anti-hepatoma activity (18, 72–74). These features make GGA a biologically relevant candidate probe for examining lipid-linked upstream events in hepatoma-cell systems. In HuH-7 cells, GGA induced concentration-dependent cell death and pyroptosis-related responses that were attenuated by pharmacological inhibition and siRNA-mediated knockdown of TLR4 (16). These findings provide functional evidence that TLR4-associated signaling contributes to the GGA response in this specific hepatoma-cell model. However, they do not establish GGA as a direct TLR4 ligand, define the proximal molecular event linking GGA exposure to receptor-associated signaling, or demonstrate equivalent TLR4 dependence across hepatoma cell lines or *in vivo* HCC. Its value in the present Perspective therefore lies in its use as a mechanistically informative perturbation through which these unresolved upstream events can be examined (16). Although GGA suppresses hepatoma cell growth and induces cell-death-related responses (23, 75–77), the proximal sequence connecting GGA exposure to inflammatory or lytic phenotypes remains incompletely understood (17). Previous studies have linked GGA exposure in hepatoma cells to stress- and cell-death-related phenotypes, including autophagy-related responses and endoplasmic reticulum stress-associated signaling (22, 23). In particular, it remains unclear whether GGA acts through receptor-associated modulation, membrane-level remodeling, intracellular stress responses, or a combination of these mechanisms. This uncertainty makes GGA less a defined signaling ligand than a useful experimental probe for linking lipid biology, innate immune signaling, and inflammatory cell-death susceptibility.

One possible mechanism is that GGA influences TLR4-associated signaling at or near the receptor-complex level. Although speculative, this model is conceptually relevant because TLR4 signaling depends not only on ligand availability but also on the organization and signaling competence of the TLR4/MD-2/CD14 receptor complex (25–27). GGA should not be regarded as a direct TLR4 agonist; however, as a lipophilic endogenous metabolite, it may influence membrane organization, receptor clustering, lipid–protein interactions, or local physicochemical conditions that regulate receptor-associated signaling (78, 79). This possibility is supported by evidence that TLR4 signaling can be shaped by cholesterol-rich membrane microdomains, raft recruitment, endocytic routing, and membrane lipid composition (80–82). More broadly, lipid species can modulate TLR4-associated inflammatory outputs, as illustrated by evidence that saturated fatty acids may promote TLR4-associated inflammatory signaling, whereas omega-3 polyunsaturated fatty acids such as EPA and DHA may attenuate TLR4 activation induced by lipopolysaccharide or saturated fatty acids (83). This broader lipid-sensitive regulation of TLR4 signaling provides a rationale for testing whether a lipophilic endogenous metabolite such as GGA can influence receptor-complex organization, membrane microenvironment, or inflammatory signaling thresholds in hepatoma cells (79, 84, 85).

A complementary possibility is that GGA acts indirectly by inducing intracellular stress and the generation or release of damage-associated molecular patterns (DAMPs) (22, 29, 30, 86). In this scenario, GGA would not primarily target TLR4 itself, but would perturb cellular homeostasis in ways that secondarily engage TLR4-associated inflammatory circuitry (86, 87). This model is compatible with hepatoma-cell biology, in which metabolic fragility, oxidative stress, and overlapping death programs frequently coexist (67, 87).

Rather, GGA is introduced here not as a conclusion, but as a hypothesis-generating experimental probe for refining the question of whether receptor-complex modulation, membrane remodeling, stress-mediated danger signaling, or their combination precedes pyroptosis-related outcomes in hepatoma cells. This working framework is summarized in Figure 2.

## 6 Experimental checkpoints to resolve the GGA–TLR4–pyroptosis interface

The working framework outlined above should be treated as an experimentally testable model rather than as an established linear pathway. Existing evidence in HuH-7 cells supports a functional contribution of TLR4-associated signaling to GGA-induced pyroptosis-related responses (16). However, this observation does not establish that GGA directly activates a linear TLR4-to-pyroptosis cascade, nor does it define the proximal event connecting GGA exposure to receptor-associated signaling. The next experimental steps should therefore focus less on a binary question of whether TLR4 participates and more on defining where, when, and under which cellular conditions TLR4-associated signaling intersects with GGA-induced stress responses and pyroptosis-related execution (16).

From an experimental standpoint, the first priority is to define the scope and position of the reported TLR4 dependence. The HuH-7 findings should be reproduced and extended across hepatoma models with distinct differentiation states and basal inflammatory phenotypes, using complementary pharmacological and genetic perturbations of TLR4-associated signaling (16, 32). Rescue experiments, where technically feasible, would further strengthen causal interpretation. Time-resolved analyses should then determine whether TLR4 perturbation alters early GGA-induced unfolded protein response, caspase-4 activation, GSDMD processing, membrane permeabilization, and lytic cell death, thereby distinguishing an upstream licensing role from secondary modulation of cell injury (16, 22, 88). Finally, direct receptor-complex engagement should be evaluated separately from indirect mechanisms involving membrane remodeling, intracellular stress, or DAMP release. These experiments would refine the reported HuH-7 association into a mechanistically ordered and model-aware framework.

A second priority is to clarify the level at which GGA acts and whether the resulting death phenotype satisfies a pyroptotic framework in mechanistic terms. If GGA acts primarily at the receptor-associated or membrane level, changes in receptor localization, clustering behavior, membrane organization, or dependence on receptor-complex components should be detectable. If GGA acts mainly through intracellular stress amplification, changes in organelle function, oxidative status, membrane integrity, or DAMP release should precede inflammatory execution (22, 29, 86, 87). Temporal analysis will therefore be essential for distinguishing mechanistic continuity from downstream convergence. In parallel, gasdermin-dependent pore formation, inflammatory caspase involvement, lytic membrane damage, and inflammatory mediator release should be evaluated directly wherever possible (89, 90). Without such validation, there is a substantial risk of conflating inflammatory injury, partial inflammasome activation, and *bona fide* pyroptotic execution under a single descriptive label.

Future studies should also address heterogeneity across hepatoma models and the relevance of the surrounding microenvironment. If the proposed framework is genuinely context dependent, different hepatoma cell lines may vary substantially in their permissiveness for GGA-induced inflammatory cell-death responses that depend on TLR4-associated signaling (67, 68). Moreover, even if GGA-induced pyroptosis-related events are demonstrated in tumor cells, their biological significance in HCC will depend on how these events influence macrophages, stellate cells, cytokine release, and fibrotic or immune remodeling at the tissue level (70, 91). Beyond the local hepatic tumor microenvironment, future studies may also consider systemic immunological readouts such as the liver–spleen axis. In metabolic liver disease, this axis has been proposed to link hepatic steatosis, chronic low-grade inflammation, insulin resistance, and spleen-mediated immune regulation (92). Although current evidence does not establish GGA as a driver of splenic TLR4-dependent pyroptosis, the spleen may serve as a downstream immunological amplifier or readout organ for evaluating systemic consequences of GGA-related modulation of inflammatory signaling. Thus, this Perspective does not presume that GGA is a confirmed upstream ligand of TLR4 or that all GGA-induced hepatoma cell death can be explained by pyroptosis. Rather, it positions GGA as a useful perturbation for testing how membrane context, danger signaling, inflammatory priming, and lytic execution may become connected in hepatoma systems. Clarifying this interface may help determine whether pyroptosis-related susceptibility conditioned by TLR4-associated priming represents a broader feature of inflammatory hepatoma biology or a context-dependent vulnerability that emerges most clearly in metabolically stressed HCC.

## 7 Discussion and conclusion

TLR4-associated inflammatory signaling and pyroptosis are both highly relevant to HCC, but their mechanistic relationship in hepatoma cells remains incompletely resolved. Current evidence supports conceptual compatibility and partial overlap more strongly than it supports a fully defined TLR4-to-pyroptosis sequence operating across HCC models. Thus, the central issue is not whether these pathways belong to the same inflammatory landscape, but under what cellular and microenvironmental conditions TLR4-associated priming becomes functionally continuous with pyroptosis-related execution.

This distinction is important because both TLR4 signaling and pyroptosis are context dependent. Inflammatory priming may contribute to chronic tissue injury, fibrogenic remodeling, and tumor-permissive microenvironmental conditioning, whereas lytic inflammatory cell death may either eliminate transformed cells or amplify a protumor milieu depending on cell type, timing, and disease stage. Therefore, the coexistence of TLR4 activation, inflammasome-related signaling, cytokine release, and loss of viability should not itself be interpreted as evidence of a unified pyroptotic mechanism. Clearer validation of pathway dependence, temporal ordering, and execution-level events remains necessary.

Within this interpretive landscape, GGA is best viewed not as a direct TLR4 ligand or a universally established regulator of pyroptosis, but as a hypothesis-generating experimental probe. Its anti-hepatoma activity and potential effects on early signaling, membrane context, and stress-related processes make it useful for testing how lipid-sensitive upstream events may intersect with inflammatory cell-death susceptibility in hepatoma-cell systems. Clarifying this interface may help determine whether pyroptosis-related susceptibility associated with TLR4 priming represents a broader feature of inflammatory hepatoma biology or a context-dependent vulnerability that emerges most clearly in metabolically stressed HCC. In this sense, the TLR4–pyroptosis relationship in HCC should be regarded not as a closed pathway, but as an open mechanistic problem that remains experimentally tractable and oncologically meaningful.

## Data Availability

The original contributions presented in the study are included in the article/supplementary material, further inquiries can be directed to the corresponding author.
